# Influence of pub Gene Expression on Differentiation of Mouse Embryonic Stem Cells into Derivatives of Ecto-, Meso-, and Endoderm in vitro

**Published:** 2009-07

**Authors:** E.V. Novosadova, E.S. Manuilova, E.L. Arsenieva, A.N. Lebedev, N.V. Khaidarova, V.Z. Tarantul, I.A. Grivennikov

**Affiliations:** 1 Institute of Molecular Genetics, Russian Academy of Sciences

## Abstract

The influence of low and high pub gene expression on the initial stages of the differentiation of mouse embryonic stem cells into derivatives of ecto-, meso-, and endoderm in vitro was investigated. As follows from the results of a RT -PCR analysis, the expression of the vimentin, somatostatin, GATA 4, and GATA 6 genes, being the markers of endodermal differentiation, does not vary in both the cells with high pub gene expression and the cells with low pub gene expression, as well as in the corresponding control lines. The cells with high pub gene expression are characterized by an increase in the expression of mesodermal differentiation gene-markers (trI card, trI skel, c-kit, and IL-7), whereas the cells with low pub gene expression are specified by a decrease in their expression. According to the analyses carried out, the reverse is characteristic of the expression of ectodermal differentiation gene-markers (nestin, ≤-III tubulin, gfap, and th). Expression of these genes decreases in cell lines with high pub gene expression, whereas their expression increases with the decrease in pub gene expression. Hence, it is suggested that the variations in the pub gene expression in the embryonic stem cells influence significantly the mesodermal and ectodermal differentiation of these cells.

## INTRODUCTION

An embryonic stem (ES) cell is a unique model to use for the investigation of the processes underway at the early stages of embryogenesis [1]. It is well known that in the course of in vivo embryo development, ES cells are able to form all three embryonic layers in culture - endoderm, mesoderm, and ectoderm - and, thus, all cell types developing from them. Analysis of gene expression in the process of ES cell differentiation into specialized cell types shows that the succession and efficiency of gene expression in the course of in vitro differentiation corresponds, as a whole, to the sequence of these processes in vivo [10]. Hence, ES cells may be used as an adequate experimental model for the investigation of molecular mechanisms at the initial stages of differentiation. Moreover, the investigations of ES cell differentiation in this or other directions in response to the action of specific inducers (growth factors, cytokines) or to direct the genetic modification of these cells make it possible to understand the functions of the investigated substances and different genes in this process [1].

In the previous investigations, we obtained and described cDNA clones characterized by intensive transcription in the HIV-associated immunoblastic lymphomas using the method of subtractive hybridization [16, 17]. An analysis of those cDNA allowed us to detect among them, along with the previously described genes (set, calpain, etc.), several cDNA coding genes with previously unknown functions. One of such lymphoma-specific genes was then termed pub. The protein product of the human pub gene (hPub) is highly homological to the mouse Pub protein (mPub) [5].

Pub is referred to the TRIM (tripartite motif) protein family [5] characterized by the presence of the so-called TRIM (or RBBC) motive composed of three Zn-binding domains such as RING (R), B-box 1 (B1), and B-box 2 (B2) accompanied by the coiled-coil (CC ) region [15]. Currently, 37 representatives of this protein family are known. Some of them are involved in such biological processes as regulation of transcription, formation of cytoskeleton, control of cell proliferation, and differentiation [18]. The functions of the hpub gene in the organism are poorly investigated. The mouse-homologue mpub plays an important role in the processes of cell differentiation and influences substantially the transcription activity of the PU.1 factor [5]. PU.1 referred to the ET S family of transcription factors plays a major role in the differentiation and proliferation of macrophages and B-cells in the course of haemopoiesis and controls the functional activity of neutrophiles [11]. The mpub gene product inhibits the transcription activity of PU.1 in hemocytes and, thus, plays a very important role in the proliferation and differentiation of myeloid and lymphoid cells [5].

The model of mouse ES cells was used to investigate the influence of the pub gene on the initial stages of their development. Earlier on, we obtained stable, transfected cell cultures with a high expression of the hpub gene (ES-hPub line) controlled by the CMV promoter, cell cultures with a low expression of the mpub gene (ES-RNAi line) caused by the action of interfering RNA, as well as the corresponding control lines (ES-DNA3 and ES-pJneo, respectively) [2]. High expression of the hpub gene resulted in an increase, while low expression of the mpub gene resulted in a decrease, in the number of embryoid bodies formed by the ES cells. However, gene expression had no influence on the proliferative activity of those cells [2, 3].

In the present paper, we estimate the influence of high and low mpub and hpub gene expression on the expression of genemarkers of ento-, meso, and ectodermal differentiation in cultures of transfected mouse ES cells using the RT-PCR method (polymerase chain reaction with reverse transcription).

## Experiments 

### Cultivation of ES cells

Mouse ES cells of the R1 line kindly provided by A. Nagy (Mount Sinai Hospital, Toronto, Canada) were used in the investigation. The ES cells were cultivated at 37°C and 5% CO2 in a ≤-MEM medium (Sigma, USA) containing 15% of fetal cow serum (FCS) (Gibco, the USA), 0.1 mM of 2-mercaptoethanol, 2 mM of L-glutamin, replaceable amino acids (Gibco, USA), nucleosides, vitamins, and gentamicin (20 ≤g/ml). Primary fibroblasts from mice 11-12 days of embryonic development in age with proliferation blocked by mitomycin C (5 ≤g/ml) were used as a feeding layer for the ES cells. A DMEM medium (Sigma, USA) containing 10% of FCS, 2 mM of L-glutamin, and gentamicin (20 ≤g/ml) was the growth environment for the primary culture of fibroblasts. When the ES cells were cultivated without the feeding layer, LIF (leukemia inhibitory factor) (Sigma, USA) in a final concentration of 10 ng/ml, which blocked the spontaneous differentiation of those cells, was added to the medium. Cell subculturing and change of the medium were carried out every 3 days.

### Induction of ES cell differentiation with formation of embryoid bodies

The ES cells were isolated from the fibroblasts of the feeding layer to induce differentiation, followed by the formation of embryoid bodies. The cells were processed with tripsin, subject to centrifuging, and then the suspension obtained was incubated in a Petri dish (d = 60 mm) (Nunc, Denmark) for 10-20 minutes in the CO2-incubator. In that period of time, most fibroblasts were attached to the dish bottom, while the ES cells remained in the suspension. To form the embryoid bodies, the suspension with the ES cells was transferred to the Petri dish (d=35 mm) (Nunc, Denmark) in a quantity of 200,000 cells per 2 ml of medium or to the 96-well immunological plate (1,000 cells per 1 well in 1 ≤l of medium) and then was placed in the CO2-incubator. On the third day of cultivation, the formed embryoid bodies were transferred to plates coated with gelatin for further differentiation.

### Extraction of total RNA and conduction of reverse transcription

Total RNA was extracted from the differentiated ES cells and other cell lines and tissues by the phenol-chloroform extraction method using the YellowSolve equipment (Clonogen, Russia) and following the manufacturer's recommendations. The procedure of reverse transcription was carried out using the Sileks equipment (Russia) in accordance with the protocol and the manufacturer's recommendations. cDNA was synthesized with the use of total RNA for 1 hour at 37°C in a 20 ≤l reaction mixture containing 0.05 ≤g of randomly selected hexaprimers, and 100 units of MMLV (moloney murine leukemia virus) reverse transcriptase. When the reaction was completed (incubation for 10 minutes at 70°C), the cDNA samples were stored at 20°C.

### Polymerase chain reaction

The polymerase chain reaction (PCR ) was carried out in a 25 ≤l reaction mixture containing Taq-buffer, 1.5 mM of dNT P mixture, 1.25 of "colored" Taq-polymerase (Synthol, Russia), 0.5 ≤l of cDNA samples, and 10 pmole of each primer. The primers selected for the corresponding genes, as well as the PCR conditions and lengths of the products, are presented in Table 1. The PCR products were separated in a 1.5% agarose gel, visualized with the help of ethidium bromide, and then analyzed using the BioDocAnalyze system (Biometra, Germany).

**Table 1:** Primers used in polymerase chain reaction.

N	Gene	Structure of primers	Annealing temperature (0C)	Number of cycles	Size of product (bps)
1	GAPDH	5'-TCCATGACAACTTTGGCATTGTGG-3'-s 5'-GTTGCTGTTGAAGTCGCAGGAGAC-3'-as	66	27	376
2	pub	5' CCCATTTGGAAGACGCCG 3' -s 5' AGGGTGGCTCAGCTCCG 3'-as	70	43	328
3	hpub	5'-GCAGCAGCACATTGACAACA-3'-s 5'-TCCACGAGGCCCTTAAAGAA-3'-as	60	30	382
7	GATA 4	5'-GGTTCCCAGGCCTCTTGCAATGCGG-3'-s 5'-AGTGGCATTGCTGGAGTTACCGCTG-3'-as	65	40	153
8	GATA 6	5'-CCGCGAGTGCGTGAACT-3'-s 5'-CGCTTCTGTGGCTTGATGAG-3'-as	65	40	137
9	trI card	5'-CCACACGCCAAGAAAAAGTC-3'-s 5'-AAGCTGTCGGCATAAGTCCT-3'-as	62	32	204
10	trI skel	5'-CACACTCTGCAGTCTGTGGTGAG-3'-s 5'-CTGAAGGGCACTGAGAGACAGAC-3'-as	64	35	314
12	nestin	5'-CGCTGGAACAGAGATTGGAAGG-3'-s 5'-GTCTCAAGGGTATTAGGCAAG-3'-as	58	30	375
13	gfap	5'-TCCTGGAACAGCAAAACAAG-3'-s 5'-CAGCCTCAGGTTGGTTTCAT-3'-as	61	42	224
14	≤-III tubulin	5'-GAGGAGGAGGGGGAGATGTA-3'-s 5'-CCCCCGAATATAAACACAACC-3'-as	65	35	348
15	th	5'-TGCACACAGTACATCCGTCA-3'-s 5'-TCTGACACGAAGTACACCGG-3'-as	60	35	376
16	vimentin	5'-ACCTGTGAAGTGGATGCCCT-3'-s 5'-AAATCCTGCTCTCCTCGCCTT-3'-as	55	30	318
17	somatostatin	5'-CAGACTCCGTCAGTTTCTGC-3'-s 5'-ACAGGATGTGAAAGTCTTCCA-3'-as	56	30	262
18	c-kit	5'-TGTCTCTCCAGTTTCCCTGC-3' -s 5'-TTCAGGGACTCATGGGCTCA-3'-as	58	45	765
19	IL-7	5'-ACATCATCTGAGTGCCACA-3' -s 5'-CTCTCAGTAGTCTCTTTAG-3'-as	57	45	355

## RESULTS AND DISCUSSION

It is known that ectodermal differentiation produces the nervous system and epithelium, endodermal differentiation produces the liver, pancreatic gland, thyroid gland, and lungs, while mesodermal differentiation produces blood, skeletal and cordular muscles (http://stem-cells.ru).

**Fig. 1.**
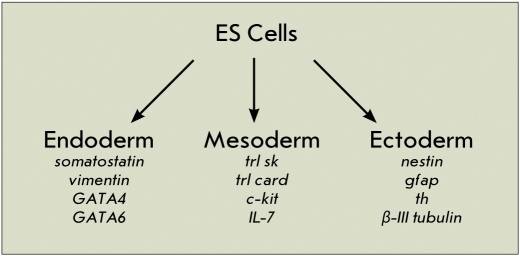
Gene-markers of the definite directions of ES cell differentiation used in experiments

We investigated the infulence of variations in mpub and hpub gene expression on the differentiation of mouse ES cells in the ectodermal, endodermal, and mesodermal directions. For that purpose we chose specific gene-markers characterizing different types of cells originating from this or another germ layer (Fig. 1).

Transfected ES cells of four lines (ES-hPub, ES-DNA3, ES-Ineo, and ES-RNAi) were subjected to "spontaneous" differentiation: i.e., we did not add any specific inductors of certain types of cell differentiation. The presence of expression and variations in its level were determined with the help of the RT-PCR method. The cells that underwent endodermal and mesodermal differentiations were analyzed on the 10th day, while the cells that underwent ectodermal differentiation were analyzed on the 21st day of cultivation [6].

### nfluence of high hpub gene expresion and low mpub gene expression on the endodermal differentiation of transfected ES cells.

GATA 4 and GATA 6 are referred to the family of transcription factors. They play a definite role in the regulation of genes involved in embryogenesis, as well as in the development of cardiovascular and viscerogenic endoderm. As follows from our investigation of the Zebrafish and Xenopus organisms, the GATA 4 and 6 genes play a very important role at the initial stages of the heart's development [7, 8, 12-14]. Moreover, the knockout of mouse GATA 4 and 6 genes causes the death of the embryo at the gastrulation stage, due to the interruption of the definitive endoderm formation [19].

**Fig. 2.**
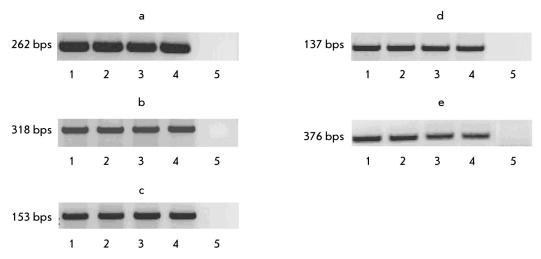
Expression of genes involved in the endodermal differentiation of stable transfected ES cells (10 days of differentiation in vitro). Data of RT-PCR analyses. Genes: a) somatostatin, b) vimentin, c) GATA 4, d) GATA 6, e) GAPDH, gene for comparison. Cell lines: 1. ES-hPub, 2. ES-DNA3, 3. ESRNAi, 4. ES-pIneo. 5. Negative control (water)

The experiments carried out established no difference in the expression of the vimentin, somatostatin, GATA 4, and GATA 6 genes in both the differentiated cells with high hpub gene expression and cells with low mpub gene expression, as well as in the corresponding control lines (Fig. 2). Hence, it can be suggested that variations in the pub gene expression do not influence the endodermal differentiation of ES cells.

### Influence of high hpub gene expression and low mpub gene expression on the mesodermal differentiation of transfected ES cells.

**Fig. 3.**
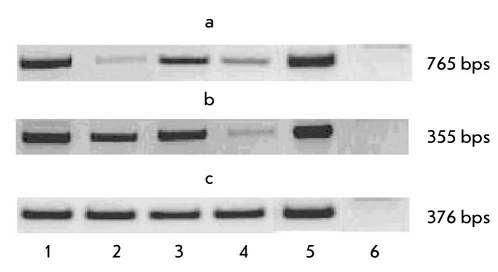
Expression of c-kit and IL-7 genes in stable transfected ES cells (10 days of differentiation in vitro). Data of RT-PCR analyses. Genes: a) c-kit, b) IL-7, c) GAPDH, gene for comparison. Cell lines: 1. ES-hPub, 2. ES-DNA3, 3. ES-RNAi, 4. ES-pIneo, 5. Positive control (mouse thymus). 6. Negative control (water)

At the following stage of the investigation, we checked the influence of hpub and mpub gene expressions on cell differentiation in the mesodermal direction. Taking into consideration the homology of mouse gene mpub and human gene hpub, we may suggest that the hpub gene product will inhibit the transcription activity of PU.1 and, by that, will influence the differentiation of hematopoetic cells. To verify that theory, we investigated the influence of high hpub gene expression and low mpub gene expression on the differentiation of ES cells in the lymphoid tissue. For that purpose, we chose specific genemarkers for lymphoid cells, such as c-kit and IL-7 [7]. On the 10th day of cultivation, a PCR analysis of transgenic cell lines showed that high hpub gene expression led to an increase in the expression of both gene markers, while inhibition of the endogenic mpub gene, on the contrary, caused a decrease in their expression as compared to the corresponding controls (Fig. 3).

Hence, the data obtained testify to the fact that high hpub gene expression may lead to ES cell differentiation through the lymphoid way.

Two genes - trI sk (skeletal troponin I) and trI card (cardiac troponin I) - were selected to determine the influence of a differently directed expression of the pub gene on ES cell differentiation into other derivative mesoderms. Both cardiac protein isoform and isoform from slow skeletal fibers are expressed in the heart of a human embryo. After birth, the expression of skeletal-muscular troponin I isoform is blocked, whereas the synthesis of cardiac isoform is stimulated [5].

**Fig. 4.**
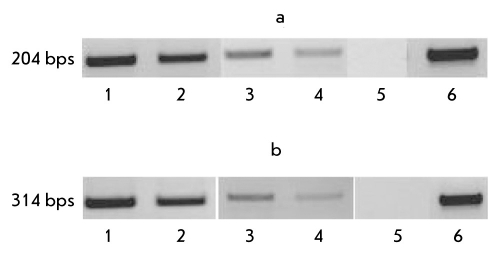
Expression of cardiac (trI card) and skeletal (trI sk) troponin I genes in stable transfected ES cells (10 days of differentiation in vitro). Data of RT-PCR analyses. a) trI card gene. Cell lines: 1. ES-hPub, 2. ESDNA3, 3. ES-pIneo, 4. ES-RNAi, 5. Negative control (water), 6. Positive control (heart of adult mouse). b) trI sk gene. Cell lines: 1. ES-hPub, 2. ES-DNA3, 3. ES-pIneo, 4. ES-RNAi, 5. Negative control (water), 6. Positive control (heart of mouse embryo)

As follows from Fig. 4, the trI card and trI sk genes are expressed in different ways. In the transfected cells with superexpression of the hpub gene, expression of these genes is also high as compared to the control line, whereas the cells with a repressed expression of the mpub gene demonstrate a lower level of expression of the troponin I genes relative to the control line.

The results obtained testify to a possible influence of the mpub and hpub genes on ES cell differentiation into different derivative mesoderms. Moreover, hpub gene superexpression leads to an increase in the level of mRNA for some gene markers of this type of differentiation.

### Influence of high hpub gene expression and low mpub gene expression on the ectodermal differentiation of transfected ES cells.

**Fig. 5.**
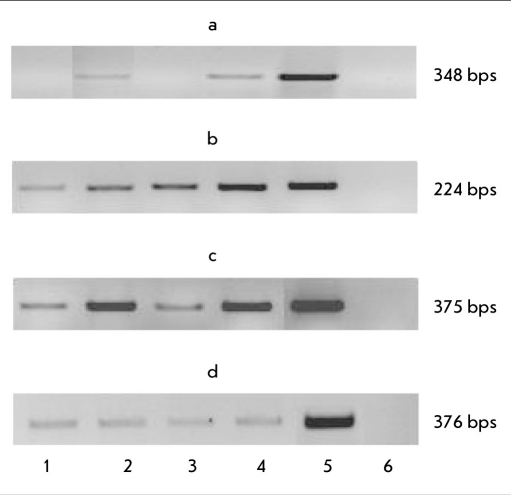
Expression of genes involved in the ectodermal differentiation of stable transfected ES cells (21 days of differentiation in vitro). Genes: a) ≤-III tubulin, b) gfap, c) nestin, d) th. Cell lines: 1. ES-hPub, 2. ES-DNA3, 3. ES-pIneo, 4. ES-RNAi, 5. Positive control (mouse hippocampus), 6. Negative control (water)

The nestin, ≤-III tubulin, gfap, and th gene markers were used to investigate the influence of the hpub and mpub genes on the ectodermal differentiation of ES cells. The nestin gene is expressed in neural stem cells, young neurons, some glial cells, and ependymal cells. The nestin gene is commonly expressed at the initial stages of formation of the central and peripheral nervous sytem. The ≤- III tubulin and gfap genes code proteins of the cytoskeleton of neurons and glial cells, respectively. Tyrosine hydroxylase gene th is expressed in dopaminergic neurons [19]. Data on the expression of these genes in the experimental and control lines of ES cells are presented in Fig. 5.

## Conclusions

As follows from the results of the PCR analysis, the expression of genes involved in the neural differentiation decreases in cells with high hpub gene expression, and the expression of these genes increases in cells with low mpub gene expression relative to the controls. This situation is characteristic of three (nestin, ≤-III tubuli, and gfap) of the four genes analyzed. Expression of the th gene does not vary in all four cell lines. This result may be explained by the fact that variations in the pub gene expression do not influence the formation of dopaminergic neurons characterized by the presence of th gene expression. Hence, according to the experiments performed, the hpub gene superexpression or the repression of mpub gene expression in mouse ES cells causes significant variations in the expression of gene markers in derivatives of some germ layers and, thus, has a definite, differently directed influence on the differentiation of cells in the meso- and ectodermal directions.

## Acknowledgements

This work was partially supported by Grants from the Ministry of Education and Science of the Russian Federation (project no. 02.512.12.2013) and the Russian Foundation for Basic Research (project no. 09-04-01117). 
